# Behavioral Treatments for Alcohol Use Disorder and Post-Traumatic Stress Disorder

**DOI:** 10.35946/arcr.v39.2.08

**Published:** 2018

**Authors:** Julianne C. Flanagan, Jennifer L. Jones, Amber M. Jarnecke, Sudie E. Back

**Affiliations:** Julianne C. Flanagan, Ph.D., is an associate professor in the Department of Psychiatry and Behavioral Sciences, Medical University of South Carolina, Charleston, South Carolina. Jennifer L. Jones, M.D., is a postdoctoral fellow in the Department of Psychiatry and Behavioral Sciences, Medical University of South Carolina, Charleston, South Carolina. Amber M. Jarnecke, Ph.D., is a postdoctoral fellow in the Department of Psychiatry and Behavioral Sciences, Medical University of South Carolina, Charleston, South Carolina. Sudie E. Back, Ph.D., is a professor in the Department of Psychiatry and Behavioral Sciences, Medical University of South Carolina, and a staff psychologist at the Ralph H. Johnson VA Medical Center, Charleston, South Carolina

**Keywords:** alcohol use disorder, comorbidity, integrated treatment, post-traumatic stress disorder

## Abstract

Alcohol use disorder (AUD) and post-traumatic stress disorder (PTSD) are highly prevalent and debilitating psychiatric conditions that commonly co-occur. Individuals with comorbid AUD and PTSD incur heightened risk for other psychiatric problems (e.g., depression and anxiety), impaired vocational and social functioning, and poor treatment outcomes. This review describes evidence-supported behavioral interventions for treating AUD alone, PTSD alone, and comorbid AUD and PTSD. Evidence-based behavioral interventions for AUD include relapse prevention, contingency management, motivational enhancement, couples therapy, 12-step facilitation, community reinforcement, and mindfulness. Evidence-based PTSD interventions include prolonged exposure therapy, cognitive processing therapy, eye movement desensitization and reprocessing, psychotherapy incorporating narrative exposure, and present-centered therapy. The differing theories behind sequential versus integrated treatment of comorbid AUD and PTSD are presented, as is evidence supporting the use of integrated treatment models. Future research on this complex, dual-diagnosis population is necessary to improve understanding of how individual characteristics, such as gender and treatment goals, affect treatment outcome.

## Overview

Alcohol use disorder (AUD) and post-traumatic stress disorder (PTSD) are chronic, debilitating conditions that commonly co-occur.^1^ The high rates of disability, physical and mental health problems, and health care utilization associated with co-occurring AUD and PTSD pose a tremendous economic burden in the United States and worldwide.^2^–^14^ Previous reviews of treatment options for comorbid AUD and PTSD indicate that effective treatments are scant, and there is substantial room for improvement.^4^–^9^ Furthermore, individuals with co-occurring AUD and PTSD suffer a more complicated course of treatment and less favorable treatment outcomes, when compared with individuals who have either disorder alone.^15^–^19^ Therefore, identifying effective interventions to treat co-occurring AUD and PTSD is a national public health priority. This review describes evidence-supported interventions targeting AUD and PTSD individually and in the context of co-occurrence.

## Behavioral Treatments for AUD

Behavioral interventions are a primary component of the treatment of AUD and can be used as freestanding treatments or as part of a more comprehensive treatment plan that includes pharmacotherapies. Behavioral interventions for AUD include providing psychoeducation on addiction, teaching healthy coping skills, improving interpersonal functioning, bolstering social support, increasing motivation and readiness to change, and fostering treatment compliance.

Cognitive behavioral therapies (CBTs) are some of the most commonly used and empirically supported behavioral treatments for AUD.^20^,^21^ Over the past 30 years, numerous meta-analyses and systematic reviews have demonstrated that CBT is an effective treatment for AUD.^20^,^22^–^25^ For substance use disorders, small but statistically significant treatment effects have been observed for various types of CBT.^24^ CBT interventions typically are designed as short-term, highly focused treatments that can be implemented in a wide range of clinical settings. These interventions are flexible and can be applied in individual or group therapy formats. CBTs for AUD focus on the identification and modification of maladaptive cognitions and behaviors that contribute to alcohol misuse.^21^ Behavioral treatments for people with AUD also target motivation for change and improvement of specific skills to reduce the risk for relapse.

Although most behavioral interventions are designed as short-term treatments (e.g., 8 to 20 sessions), many people struggling with AUD require long-term treatment. Depending on the severity of the AUD, history of treatment attempts, family history, and other risk factors, some individuals will remain in various stages of treatment for years to maintain sobriety. Furthermore, many individuals with AUD will complete several rounds of treatment and engage in several different types of treatment simultaneously (e.g., CBT and 12-step engagement). In this section, we briefly review several empirically supported behavioral interventions for AUD. (Higgins and colleagues provide more information on behavioral interventions for substance use disorders.^26^)

### Relapse prevention

For the past 30 years, relapse prevention^27^ has been one of the prevailing empirically supported CBTs for AUD.^20^ Relapse prevention is designed to help people with AUD identify high-risk situations for relapse (e.g., negative emotional states and alcohol-related cues) and develop effective coping strategies.^21^,^28^ This intervention encourages behavioral strategies such as avoiding or minimizing exposure to cues that trigger cravings, engaging in pleasant activities, and attending self-help groups. In addition, individuals receiving this treatment learn to recognize warning signs that typically precede a relapse and create a relapse management plan (i.e., an emergency plan for what to do if a relapse occurs). Relapse prevention also focuses on strategies for challenging relapse-related cognitions (e.g., “A few drinks won’t hurt”). In a review of 24 randomized controlled trials, relapse prevention was associated with reductions in relapse severity and with sustained and durable effects.^29^ Evidence from the review suggests that relapse prevention is most effective for those who have negative affect, more severe substance use disorder, and greater deficits in coping skills.

### Contingency management

Contingency management is a behavioral therapy that employs the basic behavioral principles of positive and negative reinforcement to promote the initiation and maintenance of abstinence or other positive behavior changes.^30^,^31^ The most thoroughly researched form of contingency management involves monetary-based reinforcement, in which money or vouchers can be earned and exchanged for prizes, contingent on meeting therapeutic goals.^32^ Often, the primary goal is abstinence, but other goals may include therapy attendance, prosocial behaviors, or compliance with medications.^21^,^26^ Contingency management is designed to help promote initial abstinence of substance use. This intervention can be particularly helpful when the individuals receiving treatment have little or no internal motivation, or if they lack natural reinforcers, such as family relationships.^26^,^33^ Numerous studies show that contingency management can increase abstinence, clinic attendance, and medication compliance.^32^,^34^–^37^

### Motivational enhancement

Motivational enhancement therapy is an intervention designed to enhance internal motivation for change and engagement in the change process.^38^,^39^ This therapy stemmed from the recognition that many individuals with AUD are ambivalent about changing their behavior, unmotivated, or not ready for change. Motivational enhancement therapy can be used as a stand-alone treatment or in combination with other behavioral interventions.^21^,^40^ Based on the principles of motivational interviewing,^41^ this therapeutic technique is collaborative, empathetic, and nonconfrontational. It helps individuals with AUD resolve ambivalence about quitting or reducing their alcohol intake, increase their awareness of the negative consequences of drinking alcohol and the positive benefits of abstinence, and resolve values discrepancies (e.g., valuing physical health is incompatible with alcohol misuse). Motivational enhancement therapy has been shown to be particularly effective for individuals who have AUD, for those who use nicotine, and for participants who have substance use disorder and a problem with anger.^25^,^40^,^42^–^45^

### Couples therapy

Alcohol behavioral couple therapy^46^ and behavioral couples therapy for alcoholism and drug abuse^47^ are manual-guided (also known as manualized) treatments for AUD that incorporate participation of a significant other or romantic partner. Most effective AUD treatments target individuals, but these two therapies also target relationship functioning, which is an important mechanism in the etiology, course, and treatment of AUD.^8^,^9^ Both of these therapies involve 12 weekly, 60- to 90-minute sessions that focus on psychoeducation and cognitive behavioral interventions. The interventions target relationship skills and skills related to reducing AUD severity. Alcohol behavioral couple therapy uses motivational interviewing techniques and focuses on harm reduction, and behavioral couples therapy for alcoholism and drug abuse emphasizes attaining and maintaining abstinence.

### Twelve-step facilitation

Twelve-step facilitation is a manual-guided intervention for AUD that is based on the 12 steps of Alcoholics Anonymous.^48^ Twelve-step facilitation is designed to help with early recovery and to help people engage with a local Alcoholics Anonymous or other 12-step therapy group in the community.^21^ This therapy focuses on acceptance of addiction as a chronic and progressive illness, acceptance of the loss of control over drinking, surrendering to a higher power, lifelong abstinence from alcohol, and fellowship through a group. Participants are encouraged to obtain a sponsor who will serve as a source of practical advice and support during recovery. Data from the National Institute on Alcohol Abuse and Alcoholism project Matching Alcoholism Treatment to Client Heterogeneity (Project MATCH) found that individuals who received 12-step facilitation, compared to cognitive behavioral or motivational enhancement therapies, were significantly more likely to be abstinent at follow-up visits during the 3 years after treatment.^25^ In addition, in the Project MATCH study, 12-step facilitation was found to be particularly helpful for participants whose social networks included other people who had substance use disorders.

### Community reinforcement

The community reinforcement approach is a CBT designed to enhance social, recreational, and vocational skills.^21^ Participants learn conflict resolution skills, ways to foster healthy relationships, and how to develop a new social network.^26^ This approach is different from other CBT interventions in that it targets a person’s reinforcers (e.g., family, friends, work, and hobbies) and helps reconnect that person with these sources of reinforcement.^21^ Community reinforcement is often combined with contingency management approaches to deliver external reinforcers (e.g., money) during the initial treatment period, to be followed by more natural sources of reinforcement (e.g., family and recreation) in the later stages of treatment.^26^ Treatment with disulfiram is offered as part of the community reinforcement approach to help decrease alcohol use. In addition to increasing abstinence, this approach has been shown to reduce the time spent drinking and the time spent being unemployed, away from family, and institutionalized.^26^

### Mindfulness

More recently, several mindfulness-based interventions have been developed for the treatment of substance use disorders. In general, mindfulness practices seek to redirect attention to the present moment and strengthen the development of nonattached acceptance of both pleasant and aversive experiences. One such intervention, mindfulness-based relapse prevention, builds on traditional relapse prevention.^49^ This intervention typically is delivered in an 8-week group format and includes psychoeducation regarding mindfulness and relapse, breath-focused awareness, body-scan exercise, and yoga mindfulness exercise. In one study, a mindfulness-based relapse prevention intervention resulted in reductions in heavy drinking, when compared with standard relapse prevention.^50^ The same researchers reported that the mindfulness-based approach may have yielded more enduring effects than standard relapse prevention, as evidenced by a significantly lower probability of heavy drinking at a 12-month follow-up for the participants who received the mindfulness-based intervention. However, a recent meta-analysis of nine randomized controlled trials found no differences in relapse between mindfulness-based relapse prevention and comparable interventions, such as relapse prevention.^51^

Another intervention, mindfulness-oriented recovery enhancement, is a group intervention delivered over 8 to 10 sessions.^52^ This intervention includes mindfulness training, cognitive restructuring, and savoring strategies designed to enhance positive emotions and salience of naturally occurring rewards. Less research has been conducted using this intervention, but one study found that mindfulness-oriented recovery enhancement resulted in reduced cravings and negative affect and improved positive affect.^53^

## Behavioral Treatments for PTSD

Behavioral intervention is considered a first-line approach in the treatment of PTSD. Several empirically supported behavioral interventions have been disseminated across populations and treatment settings. As with treatments for AUD, various treatment modalities for PTSD have been studied. Comprehensive analysis of the literature on this topic is challenging because of the diversity of inclusion and exclusion criteria of participants, the heterogeneous nature of PTSD symptoms, high treatment dropout rates, and symptoms that persist after treatment.^54^–^58^ Meta-analytic reviews of these treatments indicate that prolonged exposure therapy, cognitive processing therapy, and eye movement desensitization and reprocessing are among the most frequently and rigorously examined treatment options. In randomized clinical trials, these treatments all have similar levels of effectiveness.^59^–^62^ CBTs for PTSD are based on prevailing empirically supported etiological theories that suggest PTSD results from learned and exacerbated fear reactivity and disrupted cognitive and affective responses to trauma exposure.^63^ Targeting these processes in cognitive behavioral interventions typically results in substantial improvement in PTSD symptom severity^60^,^64^ and in various domains of functioning, when compared with unstructured interventions or usual treatment conditions.^65^–^67^ Treatment guidelines indicate that exposure-based psychotherapies have sufficient empirical evidence to be deemed effective PTSD treatments.^60^–^68^ These and other emerging therapies are described in this section.

### Prolonged exposure

Prolonged exposure is a manual-guided CBT consisting of 10 weekly, 60- to 90-minute individual therapy sessions.^54^ The central therapeutic component of prolonged exposure is based on Pavlovian learning theory. The treatment involves repeatedly presenting a conditioned stimulus (e.g., a trauma reminder) in the absence of an unconditioned stimulus (e.g., the traumatic event). This is accomplished through imaginal exposure during therapy sessions and through in vivo exposure in the environment. On average, prolonged exposure demonstrates robust symptom severity improvement.^69^

### Cognitive processing

Another manual-guided cognitive behavioral modality that has received strong empirical support for the treatment of PTSD is cognitive processing therapy.^70^ Cognitive processing therapy consists of 12 weekly, 60-minute individual sessions. This therapy involves creating and discussing written narratives describing the thoughts and emotions related to the traumatic event. Participants receive homework assignments designed to identify and challenge the maladaptive thought patterns that are central to the development and maintenance of PTSD symptomatology. A modified, group therapy version of cognitive processing therapy was designed and tested, with promising results.^65^ Evidence also supports the effectiveness of cognitive-only cognitive processing therapy,^71^ which includes psychoeducation about PTSD, cognitive skill-building, and learning cognitive restructuring skills. The cognitive-only therapy does not employ written narratives, and the most recent treatment manual recommends the cognitive-only therapy as the first-line version, with written narratives as an optional modification.^72^

### Eye movement desensitization and reprocessing

For the treatment of PTSD, eye movement desensitization and reprocessing has received empirical support^73^ and is one of the therapies that has received endorsement in recent U.S. Department of Veterans Affairs and U.S. Department of Defense treatment guidelines. Eye movement desensitization and reprocessing is one of the three most-studied treatments for PTSD.^59^ This therapy incorporates a variety of techniques, including prolonged exposure and cognitive restructuring, but it differs in that it applies these techniques in conjunction with guided eye movement exercises.

### Narrative exposure

Narrative exposure therapy is a manual-guided psychotherapy developed to treat PTSD among individuals seeking asylum from political or organized violence.^74^ In this technique, which also includes psychoeducation about PTSD, participants narrate their relevant developmental memories in chronological order and narrate details of their trauma exposures as they were experienced over time. Typically, the sessions are 60 to 120 minutes, approximately once a week for 4 to 10 weeks.

### Present-centered therapy

Present-centered therapy is a time-limited intervention that includes a psychoeducation component, skill development to manage daily stressors and challenges, and homework to solidify the new skills developed in sessions.^75^,^76^ This therapy has demonstrated efficacy in a variety of populations and is commonly used in randomized controlled trials as a comparator for new or adapted PTSD treatments.^77^

### Cognitive behavioral conjoint therapy

Cognitive behavioral conjoint therapy for PTSD is a manual-guided, 15-session CBT.^78^ This intervention is designed to improve PTSD symptoms and relationships at the same time. Research in this area is critical, as dyadic distress and dysfunction are saliently associated with poor individual PTSD treatment outcomes. Cognitive behavioral conjoint therapy involves psychoeducation on PTSD and relationships, learning communication skills to address avoidance related to PTSD and relationship problems, and challenging trauma-related beliefs.

### Other interventions

Additional interventions that integrate cognitive behavioral and other therapeutic approaches include emotion-focused therapy^79^ and brief eclectic psychotherapy.^80^ The empirical literature on these approaches is limited, but the research demonstrates promising findings.

## Behavioral Treatments for Comorbid AUD and PTSD

Problems with alcohol use have been included in the *Diagnostic and Statistical Manual of Mental Disorders* since its original 1952 edition, but PTSD was not introduced as a psychiatric diagnosis until the third edition in 1980.^81^ Since 1980, behavioral treatments for comorbid AUD and PTSD often have been conducted sequentially, with alcohol-first treatments being more prevalent than PTSD-first treatments. Theoretically, achievement of abstinence facilitates development of cognitive skills such as impulse control and emotion regulation. These skills are subsequently useful in trauma-focused therapies, and they help minimize the risk of alcohol use as a means of avoiding trauma processing. However, individuals with comorbid AUD and PTSD often request integrated treatment or are unwilling to stop drinking alcohol. Opponents of PTSD-first and integrated treatments voice concern that AUD symptoms will worsen if skills promoting abstinence are not well-developed first, and that PTSD symptomatology will also worsen overall.^82^–^84^

Irrespective of the theoretical debate, epidemiologic evidence suggests that integrated treatments are not yet widely used in substance use disorder treatment centers.^8^,^84^ Data from the Substance Abuse and Mental Health Services Administration (SAMHSA) *National Survey of Substance Abuse Treatment Services (N-SSATS): 2016* indicate that although 77% of the responding facilities at least “sometimes” offered some form of trauma-related counseling, only 38% reported “always or often” using this approach.^85^ This percentage has improved slightly since SAMHSA’s 2009 N-SSATS report, when 67% of respondents reported “sometimes, often, or always” offering trauma-focused treatment. In 2012, Capezza and Najavits noted that additional studies about “the content of trauma counseling currently offered by facilities” and “whether the treatment is informed by the evidence” would be useful.^86^

To better understand why integrated treatments are not used as often as sequential treatments, Gielen and colleagues conducted a qualitative study of health care provider views on treating PTSD in patients with co-occurring substance use disorder.^87^ The researchers reported that health care providers underestimate the prevalence of the comorbid conditions. Given that only 50% of substance use disorder treatment centers endorse providing a comprehensive mental health assessment, it is likely that PTSD is not systematically identified in many initial diagnostic assessments. Only 66% of substance use disorder treatment centers report offering any form of mental health treatment not related to substance misuse.^85^

Gielen and colleagues noted that health care providers frequently appreciate that comorbid AUD and PTSD are associated with more severe symptomatology and worse treatment outcomes.^87^ They also found that health care providers frequently expressed the belief that integrated treatment of AUD and PTSD would worsen cravings and reduce AUD treatment retention and efficacy. When studying the effectiveness of integrated treatments, researchers consistently use standardized therapies. However, at community substance abuse treatment centers, these therapies may not be routinely available because providers may not be trained in these approaches. Also, in some settings, providers may not be familiar with validated, standardized methods of PTSD screening. SAMHSA’s 2016 N-SSATS report did not comment on staff training levels at substance abuse treatment centers. Identifying methods to address the need for standardized treatments is an important area for future research.

Despite health care provider concerns about implementing integrated behavioral treatments for comorbid AUD and PTSD, a growing evidence base indicates that integrated treatments are safe, feasible, well-tolerated, and effective.^9^,^88^–^94^

In a recent review, Simpson and colleagues evaluated 24 randomized clinical trials (*N* = 2,294) from studies of behavioral treatments for comorbid PTSD and substance use disorder.^9^ The trials were grouped into three categories: (1) exposure-based treatments, (2) coping-based strategies, and (3) addiction-focused interventions. No significant differences in treatment retention were found across the three groups.

However, it is important to note that for the 24 trials, treatment retention measures varied widely.^9^ For example, one trial measured treatment retention as attendance at 12 out of 12 sessions, but another trial calculated the average number of sessions attended and determined that treatment was completed if participants finished at least 6 out of 25 sessions. Another trial evaluated retention based on participant provision of a urine sample at the end of 12 weeks.

Accounting for these measurement differences, participant retention for trauma-focused studies was approximately 51%.^9^ Retention was about 50% for nontrauma-focused studies and about 44% for studies that focused on substance use disorders. The trials’ control conditions had more retention than the experimental conditions, with 72% participant retention for trauma-focused studies, 53% for nontrauma-focused studies, and 31% for studies that focused on substance use disorders.

The analysis conducted by Simpson and colleagues included only a small number of studies, and more research on this topic is needed, as treatment retention among individuals with co-occurring PTSD and substance use disorder has significant room for improvement.^9^ Overall, the data indicate that trauma-focused treatments are an effective approach for reducing PTSD severity. Thus, integrated trauma-focused treatments are recommended for individuals with comorbid AUD and PTSD.^7^,^9^

Furthermore, many people report that they prefer integrated models of treatment to sequential models.^95^ Integrated treatments are linked with the self-medication hypothesis, which suggests that substances are often used as a means to manage distress associated with PTSD symptoms. Thus, integrated treatments for AUD and PTSD comorbidity have the advantages of acknowledging the interplay between AUD and PTSD symptoms and of targeting both conditions simultaneously with one health care provider and one treatment episode. The integrated model is further supported by studies indicating that PTSD symptom improvement influences subsequent AUD symptom improvement more than AUD symptom changes influence subsequent PTSD symptoms.^18^,^96^

## Integrated Behavioral Treatments

Treatment of comorbid AUD and PTSD presents substantial challenges to providers across disciplines and treatment settings. Individuals who have both AUD and PTSD demonstrate high-risk behaviors more often than those who have only one diagnosis; consequently, they require high levels of monitoring and intervention.^84^,^97^ Thus, developing effective integrated behavioral interventions to treat comorbid AUD and PTSD is a public health priority. Trials of integrated treatments demonstrate that substance use and PTSD severity decrease with the use of trauma-focused interventions, and these effects are largely maintained at 3-, 6-, and 9-month follow-ups.^98^–^100^

### Seeking safety

The seeking safety approach, a 25-session CBT focused on developing strategies to establish and maintain safety, is one of the most widely studied integrated treatments.^101^ Originally, seeking safety was designed as a group intervention, but it has also been studied as an individual format. The intervention has been shown to reduce symptoms of AUD and PTSD for a range of populations (e.g., women, men, veterans, and people who are incarcerated).^102^–^105^ Some studies showed that participants who received the seeking safety approach had better substance use outcomes than those who received treatment as usual. However, other studies found no treatment group differences for substance use or PTSD severity.^106^

The seeking safety approach, like most of the integrated treatments, does not include discussions of trauma memories or events, primarily because providers have concerns about using exposure-based practices in a group format and with people who have comorbid substance use disorder and PTSD.^107^ However, given the abundance of literature documenting the efficacy of prolonged exposure in the treatment of PTSD, development of exposure-based interventions for the treatment of comorbid AUD and PTSD has increased. A number of studies now demonstrate the safety and feasibility of employing exposure-based interventions among individuals who have PTSD and comorbid substance use disorders.^9^,^90^,^91^,^93^,^108^

### Concurrent treatment of PTSD and substance use disorders using prolonged exposure (COPE)

A manual-guided, integrated therapy that has demonstrated efficacy in treating comorbid AUD and PTSD is concurrent treatment of PTSD and substance use disorders using prolonged exposure.^109^ This therapy is a 12-session, individual intervention that synthesizes empirically validated, cognitive behavioral treatment for AUD with prolonged exposure therapy for PTSD.^110^ Several randomized controlled trials conducted in the United States and internationally demonstrate that this treatment significantly reduces AUD and PTSD severity.^96^,^100^,^111^

### Other treatments

Another cognitive behavioral approach to integrated treatment for comorbid AUD and PTSD is integrated cognitive behavioral therapy, which is a manual-guided intervention with preliminary, but growing, empirical support.^99^,^112^ This treatment consists of 8 to 12 weekly sessions for the individual and focuses on psychoeducation, mindful relaxation, coping skills, and cognitive flexibility.

Other interventions include the trauma recovery and empowerment model, which was designed for women, and a version of the same therapy designed for men.^113^ These interventions are group-based, focus on recovery skills, and have demonstrated reductions in substance use.^114^ Also, couple treatment for AUD and PTSD, a 15-session couple therapy adapted from Monson and Fredman’s cognitive behavioral conjoint therapy for PTSD,^78^ has promising preliminary empirical support.^115^

Other treatments with limited or preliminary empirical support are “transcend,” a 12-week partial hospitalization program that integrates cognitive behavioral and other theoretical approaches;^116^ the addictions and trauma recovery integrated model, an individual approach that focuses on reconstructing trauma memories;^117^ and trauma adaptive recovery group education and therapy, a group intervention designed to enhance emotion regulation.^118^ (See Table 1 for brief descriptions of the integrated treatments discussed in this section.)

## Future Research

Over the past few decades, important advances have been made in behavioral treatments for comorbid AUD and PTSD. The most notable area of progress is the development of trauma-informed, manual-guided, integrated, cognitive behavioral treatments that concurrently address symptoms of both conditions. Before these developments, sequential treatment was the only form of behavioral intervention employed. Now, individuals with comorbid AUD and PTSD, as well as their health care providers, have additional treatment options available.

For future research, it will be important to continue to advance and optimize integrated treatments and to address which individuals are ideal candidates for integrated therapies. Despite the established efficacy of integrated treatments and reported preferences for this type of therapy, treatment retention and dropout rates remain an important area of concern in this dual-diagnosis population.^99^,^100^ Further study that directly compares sequential and integrated treatment outcomes is necessary. One ongoing study addresses this gap in the literature by assessing whether retention rates between sequential and integrated treatments differ.^119^

Studies that compare other outcomes related to treatment retention and symptom improvement, such as sleep, mood symptoms, somatic medical conditions, and safety profiles (including violence and suicidality), would also be helpful. The literature currently lacks studies that examine the association between premorbid functioning and the ability to engage in manual-guided, evidence-supported therapies. Also needed is examination of how adding PTSD-focused treatment to AUD treatment will be feasible in terms of treatment costs, training requirements, and staff workload. The overlap of AUD with other substance use disorders is highly prevalent. Studies examining outcomes of integrated treatments among people with comorbid AUD and PTSD, when compared with people who have PTSD and substance use disorder involving multiple substances, is necessary to identify and target specific alcohol-related treatment needs. Finally, given the heterogeneous nature of AUD^120^ and the complex etiology, course, and treatment of both AUD and PTSD, studies that examine commonalities underlying effective behavioral treatments are essential.

Gender is another important consideration in the development of effective treatments for comorbid AUD and PTSD. Critical psychosocial and neurobiological differences between men and women have been demonstrated through research on the connection between stress (e.g., exposure to sexual trauma) and substance use disorder in the context of complex comorbidities.^121^,^122^ Also, gender may be a factor in the utilization of treatment for these conditions.^123^

Finally, individual preference is a critical consideration when matching people with treatment modalities. Emerging literature suggests that many people who have both PTSD and substance use disorder symptoms perceive a strong link between them, and they prefer integrated versus sequential treatment.^124^,^125^ Also, individuals receiving treatment might have a goal to reduce substance use rather than attain or maintain abstinence.^126^ Investigations that consider these individual and contextual factors are necessary to effectively match treatment approaches with individual needs and to maximize treatment development research for comorbid PTSD and AUD.

## Figures and Tables

**Table 1:** Empirically Supported Integrated Treatments for AUD and PTSD

Treatment	Content	Number of Sessions
**Individual Only**		
Concurrent Treatment of PTSD and Substance Use Disorders Using Prolonged Exposure^109^	Relapse prevention and coping skills integrated with prolonged exposure	12
**Individual or Group**		
Integrated Cognitive Behavioral Therapy^112^ (initially individual, then group)	Mindful relaxation, flexible thinking skills (e.g., cognitive restructuring and behavioral functional analysis)	8 to 12
Seeking Safety^101^	Coping skills, interpersonal relationship skills, self-development skills	25
Trauma Adaptive Recovery Group Education and Therapy^118^	Emotion regulation, mental focusing, executive function skills, mindfulness, interpersonal engagement and interaction skills	4 to 14
**Couples**		
Couple Treatment for AUD and PTSD^115^	Coping and relapse prevention skills, interpersonal relationship skills	15
**Group Only**		
Transcend^116^	In first half of sessions, coping skills only; trauma processing added in second half of sessions	12
Trauma Recovery and Empowerment Model^113^	Gender specific; cognitive restructuring, coping skills training, social support, communication skills	18 to 29
